# Influence of occlusal loading on peri-implant clinical parameters. A pilot study

**DOI:** 10.4317/medoral.19477

**Published:** 2013-12-07

**Authors:** Hilario Pellicer-Chover, José Viña-Almunia, Javier Romero-Millán, David Peñarrocha-Oltra, Berta García-Mira, María Peñarrocha-Diago

**Affiliations:** 1 Master of Oral Surgery and Implant Dentistry, Faculty of Medicine and Dentistry, University of Valencia, Spain; 2 Associate Lecturer in Oral Surgery on the Master’s Program in Oral Surgery and Implant Dentistry, Faculty of Medicine and Dentistry, University of Valencia, Spain; 3 Professor of Oral surgery, Director of Master’s Program in Oral Surgery and Implant Dentistry, Faculty of Medicine and Dentistry, University of Valencia, Spain

## Abstract

Objectives: To investigate the relation between occlusal loading and peri-implant clinical parameters (probing depth, bleeding on probing, gingival retraction, width of keratinized mucosa, and crevicular fluid volume) in patients with implant-supported complete fixed prostheses in both arches. Material and Methods: This clinical study took place at the University of Valencia (Spain) dental clinic. It included patients attending the clinic for regular check-ups during at least 12 months after rehabilitation of both arches with implant-supported complete fixed ceramo-metallic prostheses. One study implant and one control implant were established for each patient using the T-Scan®III computerized system (Tesco, South Boston, USA). The maxillary implant closest to the point of maximum occlusal loading was taken as the study implant and the farthest (with least loading) as the control. Occlusal forces were registered with the T-Scan® III and then occlusal adjustment was performed to distribute occlusal forces correctly. Peri-implant clinical parameters were analyzed in both implants before and two and twelve months after occlusal adjustment. Results: Before occlusal adjustment, study group implants presented a higher mean volume of crevicular fluid (51.3±7.4 UP) than the control group (25.8±5.5 UP), with statistically significant difference. Two months after occlusal adjustment, there were no significant differences between groups (24.6±3.8 UP and 26±4.5 UP respectively) (p=0.977). After twelve months, no significant differences were found between groups (24.4±11.1 UP and 22.5±8.9 UP respectively) (p=0.323). For the other clinical parameters, no significant differences were identified between study and control implants at any of the study times (p>0.05). Conclusions: Study group implants receiving higher occlusal loading presented significantly higher volumes of crevicular fluid than control implants. Crevicular fluid volumes were similar in both groups two and twelve months after occlusal adjustment.

** Key words:**Occlusal loading, crevicular fluid, peri-implant clinical parameters, T-Scan®.

## Introduction

Correct occlusion and oral hygiene are critical to the long-term success of dental implants (1). Overloading occlusion can upset peri-implant health and provoke inflammation that may lead to future peri-implant bone loss (2). Maintaining both the horizontal and vertical dimensions of peri-implant bone is essential for preserving correct soft tissue architecture and health. In a literature review, Salvi *et al.* (3) determined parameters for evaluating peri-implant health or disease; these parameters included: bacterial plaque, probing depth, bleeding on probing, keratinized mucosa width and crevicular fluid volume.

However, there is controversy as to whether peri-implant bone loss derives from occlusal overloading. While some animal studies (4-6) have associated excessive occlusal loading with peri-implant bone loss in absence of gingival inflammation, others have shown that occlusal stress does not cause peri-implant bone loss in absence (7-10) or absence (11) of gingival inflammation. The association remains unclear due, in part, to the lack of scientific evidence gleaned from human studies (12). These are difficult to design because the deliberate creation of excessive occlusal loading would be both unrealistic and unethical (13).

The aim of this study was to evaluate the relation between peri-implant clinical parameters (probing depth, bleeding on probing, gingival retraction, keratinized mucosa width and crevicular fluid volume) and occlusal overloading in patients with implant-supported complete fixed prostheses in both arches, using the T-Scan®III occlusal analysis system.

## Material and Methods

**Figure 1:**
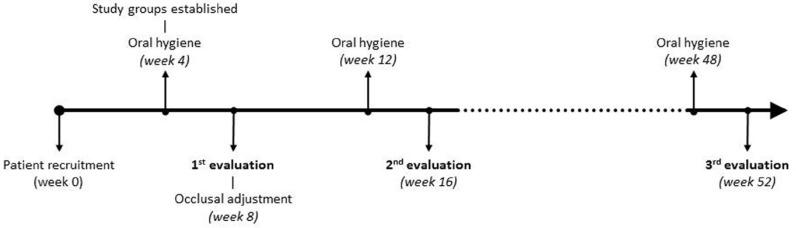
Study timeline.

-Patient selection and study design (Fig. 1)

**Figure 2:**
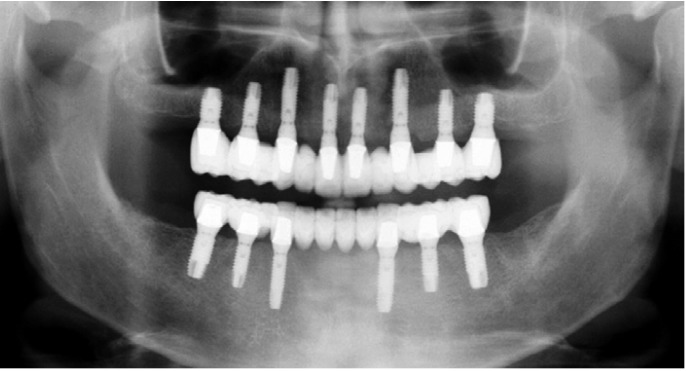
Patient rehabilitated with complete fixed prostheses in both arches.

This clinical study took place at the Oral Surgery Unit at Valencia University between October 2010 and October 2011. Fifteen patients were selected, who had been rehabilitated with ceramo-metallic complete fixed prostheses, supported by 8 Phibo TSA® implants with Avanblast surface in the upper maxillary and 6 in the mandible (Phibo Dental Solutions, Impladent, Senmenat, Barcelona, Spain) (Fig. 2).  Table 1 shows all inclusion and exclusion criteria. The study fulfilled Declaration of Helsinki principles for medical research involving humans. All patients gave their informed consent to take part and the study was approved by the University of Valencia ethics committee (ref no. H1335344280712).

**Table 1:**
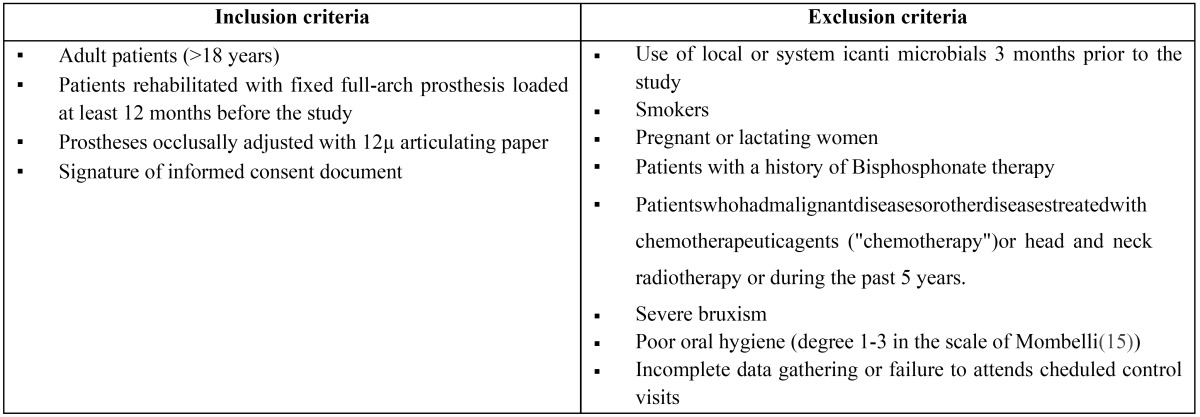
Patient inclusion and exclusion criteria.

**Figure 3:**
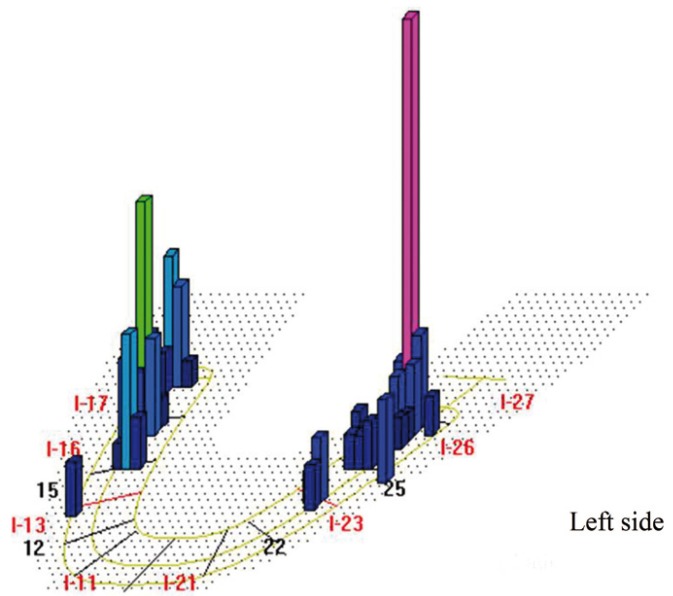
Sample T-scan III® image of occlusal contacts before occlusal adjustment (week 4).

Four patients did not fulfill the inclusion criteria: one for failing to attend scheduled appointment, 2 for poor oral hygiene and one for using mouthwashes. This left a final total of 11 patients, 4 women and 7 men, with a mean age of 58.4 years. All patients received rigorous oral hygiene with Teflon curettes and rotary instrument brushing and patients were given instructions for improving and maintaining oral hygiene at home. Patients then underwent occlusal analysis with the T-scan®III system (Tesco, South Boston, USA) in order to establish two study groups per patient: (Fig. 3) 

Study Group: Maxillary implant closest to the point of highest occlusal loading.

Control Group: Maxillary implant with least loading, furthest from the study implant.

**Figure 4:**
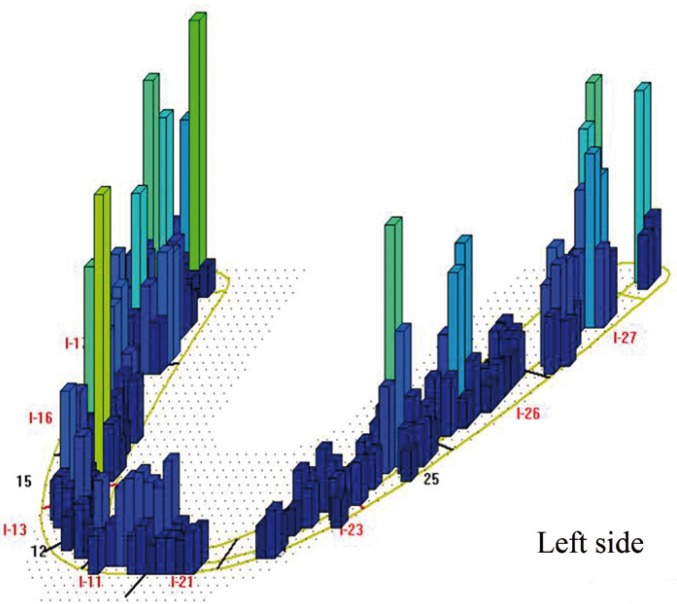
Sample T-scan III® image of occlusal contacts after occlusal adjustment (week 16).

A month later (week 8), the first set of data was collected, registering peri-implant clinical parameters (probing depth, bleeding on probing, gingival retraction, keratinized mucosa width and crevicular fluid volume). Occlusal adjustment was performed to distribute occlusal loading evenly over the whole arch, verifying the distribution with the T-Scan®III (Fig. 4), following the method described by Kerstein (14). This verification was repeated two (week 16) and twelve (week 52) months after occlusal adjustment, when peri-implant parameters were again evaluated.

-Data collection and follow-up.

Patient data were registered following a previously established protocol; in a sequence of scheduled visits to the clinic, patients were attended by a specially trained dentist, who registered the following clinical parameters:

▪ Probe depth: This was measured using a Click-Probe® plastic periodontal probe with force delivery system (Click-Probe®, Kerr, Bioggio, Switzerland). Periodontal pocket depth was measure at the selected implants, registering depth at three vestibular and lingual points and calculating the mean value.

▪ Bleeding on probing: was graded using the scale established by Mombelli *et al.* (15): grade 0= no bleeding; grade 1= isolated point bleeding; grade 2= line of blood at gumline; grade 3= profuse bleeding.

▪ Gingival retraction: was determined as the presence or absence of retraction and when present was measured at the midfacial mucosa level in relation to the edge of the prosthetic crown (16).

▪ Keratinized mucosa width: was measured in millimeters from the mucogingival line to the peri-implant groove (17).

▪ Crevicular fluid volume: crevicular fluid was collected from the implants selected for study by inserting sterile paper strips (Periopaper Strip® Proflow Incorporated. New York, NY, USA). The technique consisted of: a) air-drying the mouth; b) isolating the area with cotton wool rolls; c) gentle drying of the implant area where the paper strip was to be placed; d) crevicular fluid sample collection, inserting the Periopaper® in the groove between the implant and the gum for 30 seconds; e) placing samples between Periotron® 8000 sensors (Proflow Incorporated. New York. USA) to evaluate the quantity of crevicular fluid collected in Periotron Units (PU). The Periotron® had been calibrated previously following the manufacturer’s instructions.

-Statistical Analysis 

Statistical analysis used SPSS for windows statistical software (version 15.0. SPSS Inc., Chicago, IL, USA). Spearman and Pear-son correlation coefficients were applied to the data. Statistical significance was established as *p*<0.05.

## Results

**Figure 5:**
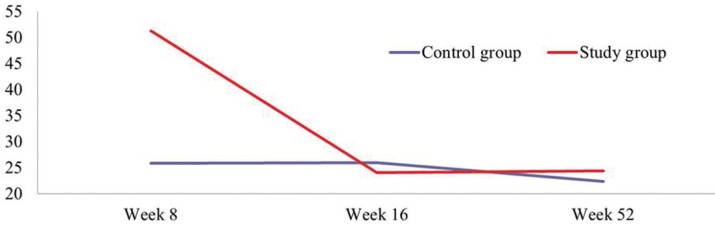
Changes in crevicular fluid volume over the three study times.

 Table 2 shows complete results for the clinical peri-implant evaluations obtained. Before occlusal adjustment, study group implants presented higher volumes of crevicular fluid than control group implants, with statistically significant difference (*p*=0.002) (Fig. 5). For the rest of the clinical parameters, no significant differences were observed between groups (*p*>0,05). At two and twelve months after occlusal adjustment, none of the clinical parameters showed any statistically significant differences between the study and control groups.

**Table 2:**
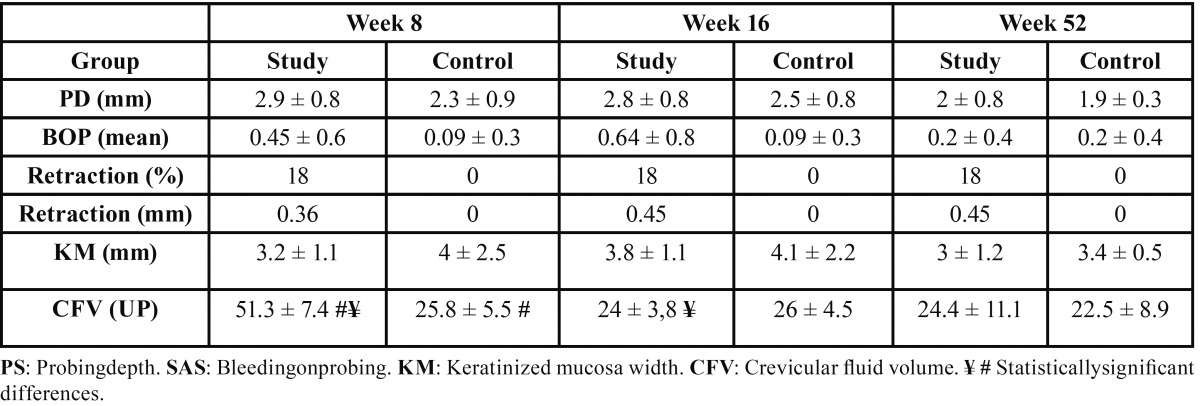
Peri-implant clinical parameters.

When results before and after occlusal adjustment were compared, a significant fall in crevicular fluid volume occurred in study group implants, resulting in almost equal volumes between the study and control groups after adjustment (*p*=0.011). These values remained stable twelve months after adjustment. However, for control group implants, no significant changes were identified (*p*=0.977). (Fig. 5) For the rest of the clinical parameters measured, no statistically significant differences were observed between study periods, before and after occlusal adjustment (*p*>0.05).

## Discussion

This study investigated the effect of occlusal loading on peri-implant soft tissues. Although the patient sample was small (11 patients, 22 implants), this represents the first study of these parameters that has used consecutive patients (selected according to strict uniform criteria and treated by the same team of dentists) and exactly the same procedures.

The clinical parameters evaluated in this study (bacterial plaque, probing depth, bleeding on probing, keratinized mucosa width and crevicular fluid volume) were selected on the basis of the literature review by Salvi *et al.* (3), which proposes these parameters for evaluating peri-implant health or disease.

Previous animal (6,9,10,18-20) and human studies (2,21,22) suggest that occlusal overloading may cause a loss of osteointegration and early implant failure. Nevertheless, the real meaning of this association is questionable due to the lack of scientific evidence obtained in studies of humans. Clearly, it would be inappropriate and unethical to monitor the effects of excessive occlusal forces in humans by deliberately placing prostheses with occlusal supra-contact. However, this has been made possible thanks to the T-scan®III occlusal analysis system, a non-invasive method, whereby the patients makes mastication movements that activate a sensor placed between the dental arches, while the computer registers and processes the data. Occlusal contacts are represented on screen by topographic images that describe the shape of the contact areas, the relative force, surface area and time sequence of occlusal contacts. Differences in occlusal loading are shown as color changes, ranging from red (high loads), graduating through the colors of the spectrum, to blue (low loads).

In all research into occlusal loading, study design is a source of controversy as it is bound to include some means of creating occlusal interference. In animal studies (5-9,18,19,23), overloading has been generated via the fixed prosthetic setup supported by splinted implants, which results in lateral overloading rather than axial. Other researchers (10,20) have created excessive occlusal loading using one-piece crowns with overloading in the antagonist arch to produce overloaded centric occlusal contacts. Indeed, Chambrone *et al.* (13) made a literature review that applied exclusion criteria to discard any studies in which implants had been splinted. But in a more recent literature review, Naert *et al.* (24) commented – and these authors agree – that research into over-loading should not be restricted to one-piece restorations. The present study used complete arch fixed prostheses so that both the study implants and the control implants formed part of a single prosthetic structure, which equalized conditions between implants.

Although differences were not statistically significant, greater probe depth was registered in implants with higher occlusal loading, a finding that coincides with other published research (6). Miyata *et al.* (6), in an experimental animal study, found that probe depth at the peri-implant groove increased with greater occlusal interference (prosthesis height of 180-µm or 250-µm compared with 100-µm). However, Kozlovsky *et al.* (20), in an experimental dog study, evaluated the impact of overloading on implants in presence or absence of inflammation; the results showed a significant increase in probe depth in implants where hygiene control had not been performed, regardless of occlusal loading conditions.

The present study evaluated bleeding on probing using the scale proposed by Mombelli *et al.* (15); implants with higher occlusal loads showed higher bleeding on probing (0.45 ± 0.6) than implants with lower loads (0.09 ± 0.3), although differences were minimal and did not reach significance. Miyata *et al.* (6,9) in two studies of similar design, observed that when the experiment period ended, occlusal overloading had not produced inflammatory responses such as redness or swelling in any of the implants studied, but in another study by the same team (19), redness and bleeding were observed in implants subjected to overloading, although the study did not include any hygiene control. Heitz-Mayfield *et al.* (10) also found bleeding on probing at 18% of over-loaded implants, but 53% of the sample presented dental plaque.

Gingival biochemical parameters and crevicular fluid volumes are determinants of current disease, patients’ susceptibility and future prognosis (25); many studies have focused on crevicular fluid volume as a potential marker for the diagnosis and prognosis of disease (26-28). Various studies have shown that crevicular fluid volume increases significantly when inflammatory conditions are present (25,29,30) and an increase in crevicular fluid volume is a useful marker of inflammation of the peri-implant and gingival tissues (25). In the present study, a decrease in crevicular fluid volumes was observed for study group implants after occlusal adjustment. To avoid the influence of oral hygiene on the periodontal parameters studied, patients received rigorous oral hygiene one month before the first data registration and were given instructions for improving and maintaining oral hygiene at home. And so, crevicular fluid volumes appeared to point to a relation between occlusal loading and the degree of peri-implant tissue inflammation. No other research has been found that has related occlusal loading with crevicular fluid volume, although Miyata *et al.* (19) observed macroscopic and histological changes (infiltration of inflammatory cells in connective tissue) in peri-implant tissue after eliminating occlusal trauma and removing plaque, concluding that the presence of inflammation and occlusal overloading could play a part in bone loss around dental implants.

Although this pilot study has its limitations, it may be concluded that implants subjected to higher occlusal forces presented significant increases in crevicular fluid volumes in comparison with implants subjected to lower occlusal loads. Two months after occlusal adjustment, when overloading had been eliminated, crevicular fluid volumes were similar in both groups. These values were stable at the twelve-month follow-up. Further research is required with longer follow-up periods and larger sample sizes to confirm these results and better evaluate the influence of occlusal over-loading on peri-implant soft tissues.

## References

[1] Kim Y, Oh TJ, Misch CE, Wang HL (2005). Occlusal considerations in implant therapy: clinical guidelines with biomechanical rationale. Clin Oral Implants Res.

[2] Uribe R, Peñarrocha M, Sanchis JM, García O (2004). Marginal peri implantitis due to occlusal overload. A case report. Med Oral.

[3] Salvi GE, Lang NP (2004). Diagnostic parameters for monitoring peri-implant conditions. Int J Oral Maxillofac Implants.

[4] Hoshaw SJ, Brunski JB, Cochran GVB (1994). Mechanical loading of Bra°nemark implants affects interfacial bone modeling and remodeling. Int J Oral Maxillofac Implants.

[5] Isidor F (1997). Histological evaluation of peri-implant bone at implants subjected to occlusal overload or plaque accumulation. Clin Oral Implants Res.

[6] Miyata T, Kobayashi Y, Araki H, Ohto T, Shin K (2000). The influence of controlled occlusal overload on peri-implant tissue. Part 3: A histologic study in monkeys. Int J Oral Maxillofac Implants.

[7] Ogiso M, Tabata T, Kuo PT, Borgese D (1994). A histologic comparison of the functional loading capacity of an occluded dense apatite implant and the natural dentition. J Prosthet Dent.

[8] Barbier L, Schepers E (1997). Adaptive bone remodeling around oral implants under axial and nonaxial loading conditions in the dog mandible. Int J Oral Maxillofac Implants.

[9] Miyata T, Kobayashi Y, Araki H, Motomura Y, Shin K (1998). The influence of controlled occlusal overload on peri-implant tissue: A histologic study in monkeys. Int J Oral Maxillofac Implants.

[10] Heitz-Mayfield LJ, Schmid B, Weigel C, Gerber S, Bosshardt DD, Jönsson J (2004). Does excessive occlusal load affect osseointegration? An experimental study in the dog. Clin Oral Implants Res.

[11] Gotfredsen K, Berglundh T, Lindhe J (2002). Bone reactions at implants subjected to experimental peri-implantitis and static load. A study in the dog. J Clin Periodontol.

[12] Klinge B, Meyle J, Working Group 2 (2012). Peri-implant tissue destruction. The Third EAO Consensus Conference 2012. Clin Oral Implants Res.

[13] Chambrone L, Chambrone LA, Lima LA (2010). Effects of occlusal overload on peri-implant tissue health: a systematic review of animal-model studies. J Periodontol.

[14] Kerstein RB (1999). Improving the delivery of a fixed bridge. Dent Today.

[15] Mombelli A, Van Oosten MAC, Schürch E, Lang NP (1987). The microbiota associated with successful or failing osseointegrated titanium implants. Oral MicrobiolImmunol.

[16] Botticelli D, Renzi A, Lindhe J, Berglundh T (2008). Implants in fresh extraction sockets: a prospective 5-year follow-up clinical study. Clin Oral Implants Res.

[17] Calvo-Guirado JL, Ortiz-Ruiz AJ, López-Marí L, Delgado-Ruiz R, Maté-Sánchez J, Bravo-Gonzalez LA (2009). Immediate maxillary restoration of single tooth implants using platform switching for crestal bone preservation: a 12-month study. Int J Oral Maxillofac Implants.

[18] Miyata T, Kobayashi Y, Shin K, Motomura Y, Araki H (1997). The influence of controlled occlusal overload on peri-implant tissue. Part 2: A histologic study in monkeys. J Jpn Soc Periodontol.

[19] Miyata T, Kobayashi Y, Araki H, Ohto T, Shin K (2002). The influence of controlled occlusal overload on peri-implant tissue. part 4: a histologic study in monkeys. Int J Oral Maxillofac Implants.

[20] Kozlovsky A, Tal H, Laufer BZ, Leshem R, Rohrer MD, Weinreb M (2007). Impact of implant overloading on the peri-implant bone in inflamed and non-inflamed peri-implant mucosa. Clin Oral Implants Res.

[21] Jofré J, Hamada T, Nishimura M, Klattenhoff C (2010). The effect of maximum bite force on marginal bone loss of mini-implants supporting a mandibular overdenture: a randomized controlled trial. Clin Oral Implants Res.

[22] Vigolo P, Zaccaria M (2010). Clinical evaluation of marginal bone level change of multiple adjacent implants restored with splinted and nonsplinted restorations: a 5-year prospective study. Int J Oral Maxillofac Implants.

[23] Isidor F (1998). Mobility assessment with the Periotest system in relation to histologic findings of oral implants. Int J Oral Maxillofac Implants.

[24] Naert I, Duyck J, Vandamme K (2012). Occlusal overload and bone/implant loss. Clin Oral Implants Res.

[25] Murata M, Tatsumi J, Kato Y, Suda S, Nunokawa Y, Kobayashi Y (2002). Osteocalcin, deoxypyridinoline and interleukin-1beta in peri-implant crevicular fluid of patients with peri-implantitis. Clin Oral Implants Res.

[26] Stewart JE, Christenson PD, Maeder LA, Palmer MA (1993). Reliability of filter-strip sampling of gingival crevicular fluid for volume determination using the Periotron. J Periodontal Res.

[27] Plagnat D, Giannopoulou C, Carrel A, Bernard JP, Mombelli A, Belser UC (2002). Elastase, alpha2-macroglobulin and alkaline phosphatase in crevicular fluid from implants with and without periimplantitis. Clin Oral Implants Res.

[28] Boynuegri AD, Yalim M, Nemli SK, Ergüder BI, Gökalp P (2012). Effect of different localizations of microgap on clinical parameters and inflammatory cytokines in peri-implant crevicular fluid: a prospective comparative study. Clin Oral Investig.

[29] Ataoglu H, Alptekin NO, Haliloglu S, Gursel M, Ataoglu T, Serpek B (2002). Interleukin-1beta, tumor necrosis factor-alpha levels and neutrophil elastase activity in peri-implant crevicular fluid. Clin Oral Implants Res.

[30] Schierano G, Pejrone G, Brusco P, Trombetta A, Martinasso G, Preti G (2008). TNF-alpha TGF-beta2 and IL-1beta levels in gingival and peri-implant crevicular fluid before and after de novo plaque accumulation. J Clin Periodontol.

